# Applying Agent-based Technologies in Complex Healthcare Environment

**Published:** 2018-03

**Authors:** Mahtab KARAMI, Ali HOSSEINI SHAHMIRZADI

**Affiliations:** Health Information Management Research Center, Dept. of Health Information Technology and Management, School of Allied-Medical Sciences, Kashan University of Medical Sciences, Kashan, Iran

## Dear Editor-in-Chief

Services in healthcare environment are carried out with knowledge-intensive agents or components as providers and consumers in order to attain one identical value. This complex integration of human-centered activities is increasingly dependent on information technology. Healthcare requires combination of many computerized systems with different user environments to provide quality efficient services in less time because it is data-intensive and technology-driven environment. The system complexities can only be dealt with methods in such environments to promote system integration and adaptation (1).

As far as software technology is concerned, agent technology as a promising research area contributes largely to the development of value-added information systems for large healthcare organizations to modify automatically themselves due to changes in their operating environment (2). Although the concept of agents first appeared in the 1970’s, however, the development of agent-based systems is today considered as a relatively new domain of software engineering. Agent locates in dynamic and complex environment as a computing entity that can autonomically sense the environment and act to accomplish its tasks or goals accordingly. The main features of the agent include reactivity, interaction and initiative autonomy, proactive and deliberative behavior, and communicative and social ability (2–6).

Healthcare environment is therefore formed of a large number of various agents in which each agent owns unique properties including behavior, data, goals, and motivations. Therefore, an agent is not full of knowledge or capacity to solve a specific problem or task. Thus, the agents must work together to accomplish goals termed Multi-Agent System (MAS). MAS is defined as the formation of a complex system as a set of entities called agents which interact to perform the system tasks (2)

Several agent-oriented methodologies (e.g., Gaia, MaSE, and MAS-CommonKADS) have been developed based on different theoretical foundations such as artificial intelligence, object-oriented programming, combination of both, and organization modeling framework (e.g., electronic Institution, OperA) (7). These methodologies are finally to contribute considerably to design complex MASs with heterogeneous agents that interact in different forms and play different roles.

The main and common components of the models are briefly defined here. Agents and Roles are predefined actions performed by an agent and then the environment constrains them according to the role it is playing. As the agents are communicating, we are then faced with concepts responsible for this communication called dialogical framework. On the other hand, the interactions that occur between the agents are referred to as scenes. A scene includes multiple agents with different roles that communicate with a performative structure. Performative structure is connectable scenes to make a network of scenes to capture the relationship among them. The performative structure describes how the agents depend on the role they are playing and can legally move from one scene to another. Normative rules are to control the interactions between the agents and ensure which is according to agreed rules (3).

Due to heterogeneity of the involved in actors; specialty, information systems, data, technology, and morbidity of patients, deliverance of healthcare services are difficult. As far as independence, adaptability, mobility, objectives, and autonomy are concerned, finding the best cooperation between them is vital. In Mobile Agent (MA) it is to overcome the problems from heterogeneous systems and offer suitable management in the permission relations in healthcare environments while the security is considered as well (4).

The technology of MA is also directed towards the internet for collecting healthcare data. MA can explore information systems among various hospitals on the internet and disperse the arithmetic processes to achieve the target. Reduced network load, network latency overcome, disconnected operation, encapsulated protocols, potential of executing asynchronously and autonomously, adapting dynamically, naturally heterogeneous, and robust and fault-tolerant are the advantages of the MA. Consequently, it is a secure and efficient method to collect electronic health records in various healthcare organizations as well as to enhance the healthcare quality (4).

In healthcare market, the segments such geriatrics health, home healthcare, telehealth, chronic disease and health-tourism can use these technologies to obtain myriad benefits such as providing pervasive and ubiquitous healthcare services for anyone from anywhere, reducing stress on the healthcare system, decreasing the cost of providing services, and monitoring the progress the rapid response to disaster (5, 6, 8–10).

## References

[1] Tien J Goldschmidt-clermont P (2009). Healthcare: a complex service system. J Syst Sci Syst Eng, 18(3):257–82.

[2] Nguyen MT Fuhrer P Pasquier-Rocha J (2009). Enhancing E-health information systems with agent technology. Int J Telemed Appl, 2009:279091. 19096509 10.1155/2009/279091 PMC2592555

[3] Rosati S Tralli A Balestra G (2013). A multi-agent system for monitoring patient flow. Stud Health Technol Inform, 192:944. 23920718

[4] Liu CH Chung YF Chiang TW (2012). A mobile agent approach for secure integrated medical information systems. J Med Syst, 36(5):2731–41. 21681511 10.1007/s10916-011-9749-3

[5] Shakshuki E Reid M (2015). Multi-agent system applications in healthcare: Current technology and future roadmap. Procedia Comput Sci, 52: 252–261.

[6] Park HS Cho H Kim HS (2016). Development of a Multi-Agent m-Health Application Based on Various Protocols for Chronic Disease Self-Management. J Med Syst, 40(1):36. 26573657 10.1007/s10916-015-0401-5

[7] Sierra C Thangarajah J Padgham L Winikoff M (2007). Designing institutional multi-agent systems. Agent-Oriented Software Engineering VII. AOSE 2006. Lecture Notes in Computer Science, vol 4405. Springer, Berlin, Heidelberg p. 84–103. https://link.springer.com/chapter/10.1007/978-3-540-70945-9_6#citeas

[8] Isern D Moreno A Sánchez D (2011). Agent-based execution of personalised home care treatments. Appl Intell, 34(2): 155–80.

[9] Hudson D Cohen M (2010). Intelligent agents in home healthcare. Ann Telecommun, 65(9–10):593–600.

[10] Sarkar A Gupta S Pramanik I Mukherje B (2012). Implementation Scheme for Online Medical Diagnosis System Using Multi Agent System with JADE. IJSRP, 2(6):1–6.

